# The Impact of Education Level on Basal Cell Carcinoma Development Risk

**DOI:** 10.7759/cureus.61827

**Published:** 2024-06-06

**Authors:** Tamari Darjania, Tina Kituashvili, George Galdava

**Affiliations:** 1 Department of Dermatology, Ivane Javakhishvili Tbilisi State University, Tbilisi, GEO; 2 Department of Dermatology, Scientific/Research National Center of Dermatology and Venereology "Kanveni", Tbilisi, GEO

**Keywords:** non-melanoma skin cancer (nmsc), basal cell carcinoma (bcc), knowledge, awareness, low education level, risk factors

## Abstract

Background: Basal cell carcinoma (BCC) stands as the most common skin malignancy, with its high incidence rate and associated costs rising annually. The origin of BCC is related to environmental, genetic, and phenotypic factors. Among these, the most important risk factor is exposure to UV light triggering keratinocyte carcinogenesis, causing cumulative cellular damage that leads to BCC development. Individuals’ educational background and awareness of skin cancer risk factors may influence the development of BCC. Lack of knowledge about risk factors (like chronic UV exposure, sunburn, artificial solar beds, and fair skin color), prevention methods, and jobs involving outdoor activities may be associated with BCC formation.

Aim: The aim of the study was to analyze recent trends and the risk factors associated with BCC, while also revealing any potential link between BCC and the patient's education level and awareness of skin cancer risk factors.

Design and methods: A hospital-based case-control study was conducted, involving a total of 141 individuals. Among them, 47 were clinically and histologically confirmed BCC patients, while the remaining participants served as controls. The control group comprised 94 individuals matched for age and gender. Data on various factors including gender, age, residency, education level, Fitzpatrick skin type, outdoor activities, use of solariums, and UV therapy, as well as awareness of potential BCC triggers, were collected using an adapted questionnaire and subjected to analysis. The collected data underwent statistical evaluation.

Results: Most of the BCCs (n = 52; 71.2%) were located in sun-exposed areas (p < 0.001), with a female/male ratio of 1.35 to 1. The nodular type of BCC was the most common form (n = 49; 67.2%). The percentage of patients in the study group with Fitzpatrick phototypes I and II (n = 38; 80.9%) was significantly higher than in controls (n = 50; 53.2%, p = 0.002). The percentage of persons with higher education levels (bachelor's degree, master's degree, and post-diploma) was significantly less prevalent among cases compared to controls (n = 20 (42.6%) vs. n = 58 (61.7%), respectively (p = 0.033)). Notably, BCC patients with low education levels exhibited significantly lesser awareness concerning genetic factors and chronic solar radiation.

Conclusions: Coexistence of factors, such as a medical history of skin cancer, having Fitzpatrick skin types I and II, engaging in outdoor work exposed to the sun, knowledge that genetic factors are risk factors of skin cancer, and knowledge that stress is a risk factor of skin cancer, are significant predictors of the disease. A lower level of education and limited awareness about risk factors can also be a risk factor for BCC. It is essential to raise awareness about potential triggers and preventive measures within the population to reduce the incidence of the disease.

## Introduction

Basal cell carcinoma (BCC) stands as the most common slow-growing keratinocyte skin cancer accounting for approximately 70% out of all non-melanoma skin cancers (NMSCs), followed by squamous cell carcinoma (SCC) with 25% [1]. It is the most commonly diagnosed skin cancer in fair-skin-colored people. Worldwide, the incidence of NMSCs varies widely, with the highest rates in Australia (>1000/100 000 person‐years for BCC) and the lowest rates in parts of Africa (<1/100 000 person‐years for BCC), and the incidence is rising annually worldwide by almost 10% each year [2,3]. BCC arises from epidermal basal cells and has a low metastatic potential (0.0028% to 0.5%); however, untreated BCC tends to be aggressive and locally invasive and can cause the destruction of the skin and underlying tissues [4]. BCC can appear anywhere on the body; however, the most common locations are sun-exposed areas, such as the face, neck, and decollate, followed by the trunk and extremities [5]. Classification of clinical variants of BCC varies by literature data, and there is no single classification of the disease. However, we can distinguish more common clinical variants of BCC including nodular, micronodular, superficial, morphoeic, keratotic, ulcerative, fibroepithelial (fibroepithelioma of Pinkus), and pigmented [6]. Each variant differs by clinical presentation, histopathology, and aggressive behavior [5].

In the last few years, the interest in the etiology and risk factors of BCC has grown, mainly due to the increased incidence of the disease and the depleting ozone layer [7]. The etiology of BCC is a multifactorial combination of phenotype, genotype, and environmental factors. It is more common in people of the Caucasian race and with genetically predisposed Fitzpatrick skin types I and II [8]. Long chronic exposure to ultraviolet radiation, particularly UVB, was already clarified to be the most important risk factor for BCC [9]. The World Health Organization and the International Agency for Research on Cancer have already classified UV radiation as a class 1 human carcinogen, as it induces keratinocyte carcinogenesis with cumulative cellular damage leading to BCC development after several years [10]. Engaging in certain jobs or hobbies could contribute to the development of BCC, according to several studies. These studies suggest that individuals who regularly participate in outdoor activities and are exposed to UV radiation over long periods are at a greater risk of developing BCC and other NMSCs in general [11]. In addition, artificial UV radiation (tanning beds), ionizing radiation, or exposure to other environmental carcinogenic agents may increase the risk of BCC in some cases. Other possible risk factors of BCC are a family history of skin cancer, some genetic disorders, previous treatments with radiotherapy or phototherapy, immunosuppression, organ transplant, and exposure to arsenic [12].

Several studies have shown that high socioeconomic status, measured by education and income, can be strongly associated with an increased risk for BCC, and this risk could be related to increased UV exposure from frequent sun-seeking trips in more wealthy regions [13,14]. These data suggest the hypothesis that BCC is becoming the disease of rich people [15]. However, a lot of campaigns against UV radiation and tanning beds increased awareness about skin cancer, and nowadays more and more people are using sunscreens for skin protection and prevention. On the other hand, people with low socioeconomic status, whose jobs are related to rural agriculture and who must be chronically exposed to the sun, are also at a high risk of development BCC as they are less likely to seek shade and less likely to use sunscreen creams [16]. The difference between these two opinions in the literature and the different lifestyles of people worldwide makes this issue more interesting and the subject of further investigation.

Our study aimed to analyze the recent clinical trends and risk factors associated with BCC and reveal the potential relationship between BCC and patient education level and awareness about skin cancer risk factors.

## Materials and methods

A hospital-based case-control study was conducted at the Dermatology Department of the Scientific/Research National Center of Dermatology and Venereology "Kanveni" in Tbilisi, Georgia, during the period of 2019-2023 by the study protocol approved by the Ethics Committee of the Center (approval no. 18/6, date 20/09/2018). The inclusion criteria of the study were patients with clinically and histologically proven BCC who gave consent. The exclusion criteria involved patients with genetic disorders like basal cell nevus syndrome (Gorlin-Goltz syndrome), xeroderma pigmentosum, nevus sebaceous, epidermodysplasia verruciformis, Rombo syndrome, Bazex syndrome, and albinism. In addition, the exclusion criteria were patients with psychosomatic issues, those unable to independently complete the questionnaire (disability of reading, writing, and understanding the questions), individuals who underwent photo and radiotherapy, and those with chronic immunosuppression.

Based on the above-mentioned inclusion and exclusion criteria, 47 patients were selected as cases. The control group, consisting of 94 participants, included the patients who visited our clinic with other non-cancerous dermatoses. They were carefully chosen to match the age and gender of the cases. Thus, the ratio between cases and controls was 1:2.

The diagnosis of BCC in all patients was initially clinical, followed by dermatoscopy and histological confirmation. After obtaining informed consent, the patients were asked to complete an anonymous questionnaire. The instrument used in the study was an adapted version of a self-administered questionnaire designed to quantify the risk for skin cancer, validated by Mexican dermatologists, along with a questionnaire used in the Euromelanoma Campaign [17,18]. The questionnaire obtained information regarding gender, age, residence, education level, Fitzpatrick skin type, sun-exposure behavior, outdoor activities, use of solariums, UV therapy, and finally awareness regarding the possible triggers of BCC. Separate questionnaires were filled out for each patient, documenting the medical history of the disease, clinical forms, and distribution of BCC lesions.

Patients were divided into two subgroups according to their education level. Subgroup 1 included patients with lower education levels (primary school, secondary school, and college). Subgroup 2 included patients with higher education levels (bachelor's degree, master’s degree, and post-diploma). We classified BCC clinical subtypes as nodular, superficial, morphoeic, pigmented, and fibroepithelial.

Statistical analysis of the obtained data was performed using the IBM SPSS Statistics for Windows, version 22.0 (released 2013, IBM Corp., Armonk, NY). Continuous variables are presented as mean ± standard deviation (SD). The difference between these parameters of the study and control groups was assessed by a two-sided t-test and Fisher’s exact test. Categorical variables are presented as percentages. The difference between these parameters of the study and control groups was evaluated by a chi-square test and Fisher’s exact test. The odds ratio (OR) and its 95% confidence intervals were used to evaluate the risk factors of the disease.

A multiple regression analysis was conducted to evaluate the combined significant impact of various social and environmental risk factors and co-factors (employment, family status, education level, residence, skin color, hair color in youth, eye color, cancer history, sun impact, smoking, and knowledge of skin cancer risk factors). The outcome variable was the presence of a disease. The criterion for rejecting the null hypothesis was p < 0.05.

## Results

Among the 47 patients with BCC enrolled in the study, 27 (57.4%) were women and 20 (42.6%) were men (mean age 67.2 ± 12.5, median = 71 years, varying from 32 to 88 years old). The female-to-male ratio was 1.35 to 1. There were eight patients (17.02%) with personal and/or family history of BCC. Seven patients (14.9%) had multiple BCC at the time of the consultation and the number of tumors varied between two and 18.

In terms of tumor location, most lesions were localized on the head, with the face being the most affected area. The distribution of tumors by sites is presented in Table 1. The most prevalent area is the nose (n = 18; 24.6%), followed by the waist (n = 9; 12.3%), periorbital area (n = 7; 9.6%), and forehead (n = 6; 12.8%). Most of the BCCs (n = 52; 71.2%) were in the sun-exposed areas, revealing a statistically significant association (chi^2 ^= 3.16, df = 1, p < 0.001). Of the non-sun-exposed skin areas, a high percentage of cases were found on the waist (n = 9; 12.3%) and on the abdomen (n = 4; 5.5%).

**Table 1:** Distribution of tumors (n = 73) by sites.

Localization	n	%
Nose	18	24.6%
Waist	9	12.3%
Periorbital area (eyelids)	7	9.6%
Forehead	6	8.2%
Cheek	5	6.8%
Perioral area	5	6.8%
Scalp (parietal part)	4	5.5%
Ear	4	5.5%
Abdomen	4	5.5%
Shoulder	3	4.1%
Eyebrow	2	2.7%
Clavicle area	2	2.7%
Chest	2	2.7%
Hip	1	1.4%
Upper arm	1	1.4%
Chi^2^ = 36.22, df = 14, p = 0.001		

Out of the total 73 tumors, the nodular subtype of BCC was the most common form (n = 49, 67.2%), followed by pigmented (n = 16, 21.9%), superficial (n = 5, 6.8%), and morphoeic (n = 3, 4.1%) forms. Based on the Fitzpatrick skin type scoring scale, the participants considered as cases were presented with Fitzpatrick phototype I (n = 6, 12.8%), phototype II (n = 32, 68.1%), type III (n = 8, 17.0%), and type IV (n = 1, 2.1%). Out of 94 controls, six (6.9%) were Fitzpatrick phototype I, 44 (46.8%) were phototype II, 33 (35.1%) were phototype III, and 11 (11.7%) were phototype IV. The difference between the distribution of phototypes in the groups was significant (chi^2^ = 11.03, df = 3, p = 0.012). The obtained results showed that the percentage of patients in the study group with Fitzpatrick phototype I and II (n = 40, 80.9%) was significantly higher than in controls (n = 50, 53.2%; OR = 3.71; 95%CI = 1.62-8.54; p = 0.002). The mentioned data are presented in Table 2.

**Table 2:** Distribution of participants according to the Fitzpatrick skin phototype.

Answer	Cases (n = 47)		Controls (n = 94)		Chi^2^ test	p
	n=	%	n=	%		
Phototype VI	0	0%	0	0%	N/A	N/A
Phototype V	0	0%	0	0%	N/A	N/A
Phototype IV	1	2.1%	11	11.7%	3.66	0.056
Phototype III	8	17.0%	33	35.1%	4.93	0.026
Phototype II	32	68.1%	44	46.8%	5.66	0.017
Phototype I	6	12.8%	6	6.4%	1.63	0.202

**Figure 1:**
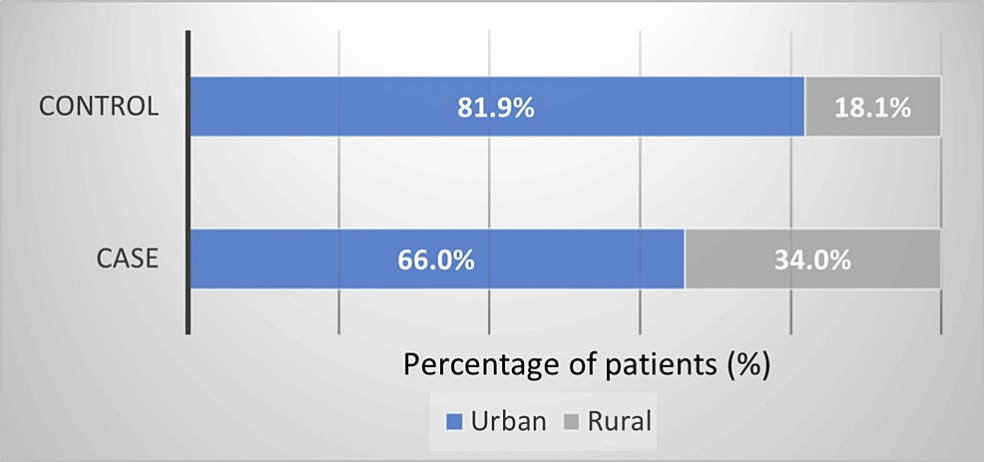
Distribution of participants by residence (urban/rural) in the study (n = 47) and control (n = 94) groups.

The distribution of participants by the residence (urban/rural) in the study and control groups is presented in Figure 1. The percentage of rural residents was significantly higher in cases (n = 16, 34.0%) versus controls (n = 17, 18.1%) (chi^2^ test = 2.59, p = 0.037, OR = 2.34) (95%CI = 1.05-5.20, p = 0.038).

The distribution of participants by education level is presented in Table 3. The percentage of persons with higher education levels (bachelor's degree, master’s degree, and post-diploma) was significantly lower in the cases (n = 20, 42.6%) versus controls (n = 58, 61.7%) (chi^2^ test = 9.56, p = 0.044). OR was 2.18 (95%CI = 1.07-4.43, p = 0.033).

**Table 3:** Distribution of participants by education level in the study and control groups.

Education level	Cases (n = 47)		Controls (n = 94)		Chi^2^ test	p
	n=	%	n=	%		
Primary school	1	2.1%	3	3.2%	0.13	0.721
Secondary school	16	34.0%	14	14.9%	6.81	0.009
College	10	21.3%	19	20.2%	0.02	0.883
Higher education	20	42.6%	58	61.7%	9.56	0.044

**Figure 2:**
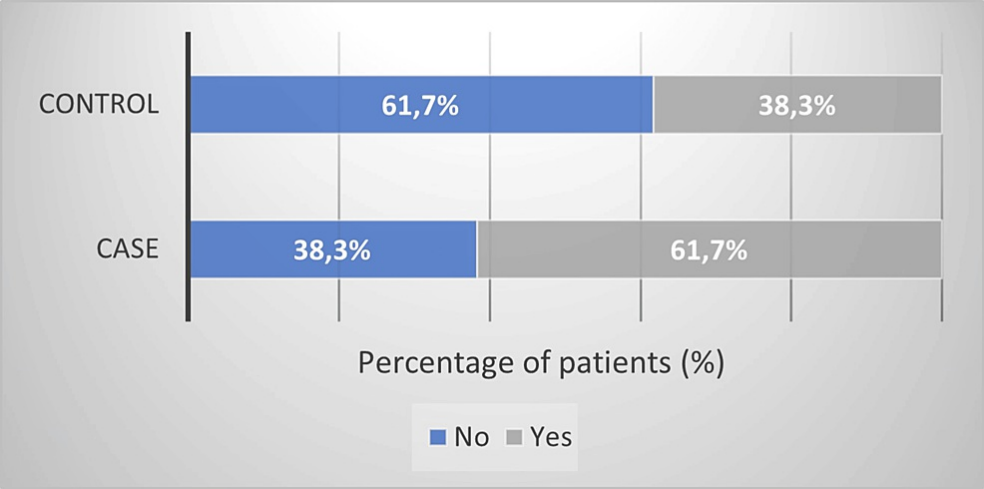
Distribution of the answers to the question regarding a job that involved working outside in the sun in the study (n = 47) and control (n = 94) groups.

One of the questions was about having a job that involved working outside in the sun. The distribution of dichotomous answers (No/Yes) in the groups is presented in Figure 2. The percentage of patients with this kind of job in the study group (n = 29, 61.7%) was significantly higher than in controls (n = 36, 38.3%) (OR = 3.27, 95%CI = 1.58-6.78, p = 0.001).

To assess the knowledge about the risk factors of skin cancer, the participants filled in a simple questionnaire by ticking dichotomous answers (No/Yes) for the following possible factors: diet, genetics, sun burning, watching TV, chronic sun exposure, mobile phones, stress, smoking, skin trauma, and artificial sunbeds (solarium). The obtained results (positive answers) and corresponding OR are given in Table 4. The patients in the study group showed significantly lower awareness regarding genetic factors and the hazards associated with solarium use. However, their knowledge about other factors was more or less comparable.

**Table 4:** Distribution of positive answers to the question regarding the risk factors of skin cancer.

Risk factor	Cases (n = 47)		Controls (n = 94)		OR (95%CI)	Chi^2^ test (p)
	n=	%	n=	%		
Genetics	14	29.8%	60	63.8%	0.24 (0.11-0.51)	14.46 (<0.001)
Artificial sunbed (solarium)	21	44.7%	69	73.4%	0.29 (0.14-0.61)	11.12 (0.001)
Sun burning	17	36.2%	49	52.1%	0.52 (0.25-1.07)	3.18 (0.075)
Mobile phones	4	8.5%	14	14.9%	0.53 (0.16-1.71)	1.14 (0.286)
Skin trauma	13	27.7%	39	41.5%	0.54 (0.25-1.15)	2.56 (0.110)
Diet	2	4.3%	5	5.3%	0.79 (0.14-4.24)	0.07 (0.785)
Smoking	13	27.7%	29	30.9%	0.85 (0.40-1.86)	0.15 (0.697)
Chronic solar radiation	27	57.4%	51	54.3%	1.13 (0.56-2.31)	0.13 (0.720)
Watching TV	4	8.5%	6	6.4%	1.36 (0.37-5.09)	0.21 (0.644)
Stress	20	42.6%	27	28.7%	1.84 (0.88-3.81)	2.68 (0.102)

To assess the impact of education level on the awareness of the patients, the study group was divided into two subgroups: subgroup 1 (lower education levels) or the patients who graduated primary, secondary schools, and college (n = 27), and subgroup 2 (higher education levels) or the patients with bachelor, master’s degree and post-diploma (n = 20). The obtained results (positive answers) and corresponding OR are given in Table 5. Patients with lower education levels demonstrated significantly less awareness regarding genetic factors and chronic solar radiation. Conversely, awareness levels regarding other factors appeared relatively consistent across both groups.

**Table 5:** Distribution of the positive answers to the question regarding the risk factors of skin cancer among cases divided by the education level.

Risk factor	Subgroup 1 (n = 27)		Subgroup 2 (n = 20)		OR (95%CI)	Chi2-test (p)
	n=	%	n=	%		
Genetic	4	14.8%	10	50.0%	0.17 (0.04-0.69)	6.656 (0.010)
Chronic solar radiation	12	44.4%	15	75.0%	0.27 (0.08-0.95)	4.295 (0.038)
Sun burning	8	29.6%	9	45.0%	0.52 (0.15-1.72)	1.151 (0.283)
Watching TV	2	7.4%	2	10.0%	0.72 (0.09-5.60)	0.097 (0.755)
Mobile phones	2	7.4%	2	10.0%	0.72 (0.09-5.60)	0.097 (0.755)
Stress	11	40.7%	9	45.0%	0.84 (0.26-2.70)	0.083 (0.772)
Artificial sunbeds (solarium)	12	44.4%	9	45.0%	0.98 (0.31-3.13)	0.001 (0.970)
Skin trauma	8	29.6%	5	25.0%	1.26 (0.34-4.66)	0.121 (0.729)
Smoking	8	29.6%	5	25.0%	1.26 (0.34-4.66)	0.121 (0.729)
Diet	2	7.4%	0	0.0%	N/A	N/A

Multiple regression analysis showed that the model reveals a moderate collective effect of variables (risk and co-factors) on the outcome (manifestation of skin cancer). Results of the multiple linear regression indicated that there was a moderate collective significant effect of the skin cancer in anamnesis, Fitzpatrick skin type I and II, having a job that involved working outside in the sun, knowledge that genetic factors are risk factors of skin cancer, and knowledge that stress is a risk factor of skin cancer on the outcome (F-test (5, 135) = 14.02, p < 0.001, r = 0.58, R^2^ = 0.34, R^2^_adjusted_ = 0.32).

The individual predictors were examined further, and they indicated that skin cancer in medical history (t = 3.21, p = 0.002), Fitzpatrick skin types I and II (t = 3.33, p = 0.001), having a job involving working outside in the sun (t = 3.71, p < 0.001), knowledge that genetic factors are risk factors of skin cancer (t = 3.87, p < 0.001), and knowledge that stress is a risk factor of skin cancer (t = 3.02, p = 0.003) were significant predictors in the model.

None of the study cases or controls had a history of treatment with UVA and UVB therapy. In addition, none of the patients had been exposed to ionic radiation or chemicals, undergone immunosuppressive treatment, or used artificial tanning beds even once.

## Discussion

BCC stands as the most prevalent form of skin cancer among humans. While global studies typically point to its higher incidence in men than women [1,2], our research showed a different finding: it was 1.35 times more prevalent in women than in men. This variance may be attributed to the prevalent general tendency among women, particularly in our region, to pursue tanning, especially during summer vacation.

BCC can manifest anywhere on the face and body, although it typically emerges more prominently in areas exposed to the sun, especially the face [5]. In this study, we found that the face was the most commonly affected area, with frequent occurrences on central facial sites, such as the nose, forehead, periorbital, and perioral regions. This again reflects the importance of solar radiation as a leading causative factor in BCC development.

There are multiple clinical types of BCC, and the most common subtype is nodular, which appears as a shiny, pearly papule or nodule with arborizing small telangiectasias; this form is more characterized by ulceration and bleeding. Superficial BCC is presented as a thin, scaly, red macule, patch, or plaque and is the second most common clinical form of BCC and usually appears on the trunk. Sclerosing/morphoeic BCC presents as pink to ivory-white, scar-like, smooth indurated plaque and accounts for approximately 5-10% of all forms [5,6]. Every mentioned subtype of BCC can have pigmentation. In some literature, pigmented BCC is a separate clinical subtype [6]. The patients enrolled in our study were presented by nodular, superficial, pigmented, and morpheaform BCCs. The most common type was nodular, followed by pigmented, superficial, and morpheaform.

The occurrence of BCC is influenced by individual susceptibility and skin type. This implies that individuals with lower melanin levels in their skin, who tend to burn rather than tan when exposed to ultraviolet radiation, are at higher risk. The Fitzpatrick skin type serves as a reliable indicator of BCC risk among people with white skin. In the case group, participants classified as Fitzpatrick phototypes I to IV were studied, revealing a higher prevalence of the disease among those with fairer skin tones (types I and II). The data mirror findings in the literature, indicating a higher incidence among individuals of the white race. This disparity may be attributed to the protective nature of darker skin against the harmful effects of UV radiation, primarily due to higher melanin levels and its protective properties [19]. Interestingly, none of the patients reported using tanning beds, a trend that can be linked to their surge in popularity about 15 years ago, particularly among millennials. In addition, it is noteworthy that nearly 98% of all participants in our study had never used sunscreen in their lifetime.

Clearly, chronic sun exposure stands out as one of the primary risk factors for developing BCC. UVB radiation directly damages cellular RNA and DNA, leading to the formation of covalent bonds between adjacent pyrimidines and the production of mutagenic products. In addition, UVA triggers the formation of toxic reactive oxygen species. Furthermore, UV exposure suppresses the cutaneous immune system in a dose-dependent manner, impairing the local antitumor-monitoring activity of dendritic cells [20]. In our current study, we discovered a significant association between chronic sun exposure and outdoor activities with the development of BCC.

A nationwide cohort study by Steding-Jessen et al. exploring socioeconomic status and NMSC revealed that high socioeconomic status, measured by education and disposable income, was strongly associated with a higher risk for BCC [13]. This could be attributed to people with high socioeconomic status being frequently exposed to UV radiation because of frequent trips to sunny resorts. However, our study showed that BCC was less prevalent among people with high education levels, and compared to the controls, they were twice as likely to develop the disease. The dichotomous answers assessing the awareness of the study groups regarding possible BCC risk factors in our study showed that controls exhibited a greater understanding of genetic factors, sunburn, and tanning beds. Responses regarding other potential risk factors did not differ significantly between the two groups. In addition, the cases with higher education levels demonstrated greater awareness of genetic factors and chronic sun exposure as triggers for BCC compared to those with low education levels. This shows how important it is to educate all groups of individuals.

The limitation of the present study is the small sample size. This limitation is compounded by the difficulty in accessing patients with BCC as the study was conducted during the COVID-19 pandemic. In addition, the absence of comprehensive information pertaining to the current residence status (urban or rural) of the participants poses a challenge. During the sample collection phase, few study subjects mentioned that they had changed their domiciles between urban and rural. Regrettably, due to prior approval from the ethics committee and the already established questionnaire protocol, amendments to address this issue were not feasible. It is imperative that future researchers bear these limitations in mind during their research.

## Conclusions

BCC is a disorder with a complex and multifactorial origin. Many possible triggers and risk factors influence its development. Significant predictors of the disease include the coexistence of factors like skin cancer in medical history, having Fitzpatrick skin types I and II, a job involving work outside in the sun, knowledge that genetic factors are risk factors for skin cancer, and acknowledgment of stress as a risk factor for the disease.

Individually, a low educational status can also be a risk factor. This is often because individuals in this group may lack knowledge about the risk factors associated with BCC. Moreover, their occupations (such as agricultural work) often entail increased exposure to UV radiation.

Educating patients, especially the susceptible population, about the risk factors of BCC, teaching them how to recognize early-stage BCC lesions through self-examination, and providing information about the ways of prevention, are crucial steps in detecting and preventing the disease at early stages.
